# Occult HBV Infection in Immunized Neonates Born to HBsAg-Positive Mothers: A Prospective and Follow-Up Study

**DOI:** 10.1371/journal.pone.0166317

**Published:** 2016-11-11

**Authors:** Ying Lu, Ya-Lin Liu, Jing-Jing Nie, Xiao-Feng Liang, Ling Yan, Fu-Zhen Wang, Xiang-Jun Zhai, Jian-Xun Liu, Feng-Cai Zhu, Zhan-Jun Chang, Jie Li

**Affiliations:** 1 Department of Microbiology and Center of Infectious Disease, School of Basic Medical Sciences, Peking University Health Science Center, Beijing, 100191, China; 2 Chinese Center for Disease Control and Prevention, Beijing, 102206, China; 3 Department of Infectious Disease, Jiangsu Provincial Center for Disease Control and Prevention, Nanjing, 210009, China; 4 Department of Major Projects, Zhengzhou Municipal Center for Disease Control and Prevention, Zhengzhou, 450053, China; Singapore Institute for Clinical Sciences, SINGAPORE

## Abstract

**Objective:**

Occult HBV infection (OBI) has been reported in infants born to HBsAg-positive mothers despite immunization. This study aims to determine the maintenance of this status in a prospective birth cohort.

**Methods:**

A total of 158 neonates born to HBsAg-positive mothers were enrolled. All received passive-active immunization against HBV according to a 0-1-6 schedule. Sera were collected at 7 months of age. Those diagnosed with OBI were serially followed up at 12, 24 and 36 months of age. HBV serological markers were determined by Abbott i2000 system. HBV DNA was quantitated by Abbott m2000 system. Standard PCR followed by direct sequencing were applied for mother-child HBV pairs. Homology and phylogenetic comparisons were done by BLAST and Mega 5.

**Results:**

All the 158 neonates were HBsAg-negative and anti-HBs-positive at 7 months of age, and 32 (20.3%) of them were diagnosed with OBI, with a median HBV DNA level of 1.97 (1.20–3.71) log IU/mL. Of them, HBV DNA was positive in 25.0%, 21.9% and 7.7% at 12, 24 and 36 months of age, respectively. HBV DNA disappeared at one of the follow-up points in 31 neonates, however, rebounded to low levels in 6 of them thereafter. HBV DNA persisted at low levels during follow-ups in the other one neonate apart from the above 31. All remained negative for HBsAg. Only two (6.3%) neonates were positive for anti-HBc after 24 months of age. HBV showed close homology and phylogenetic relationships for mother-child pairs. S-escape mutant, G145R, was not discovered. The first vaccine dose within 6 hours of birth significantly reduced the occurrence of OBI (59.4% vs. 83.3%, *p* = 0.003).

**Conclusions:**

HBV may be controlled in immunized neonates of HBsAg-positive mothers, after being diagnosed with OBI. Timely vaccination against HBV may provide the utmost protection. Long-term and close monitorings are needed.

## Introduction

Occult hepatitis B virus (HBV) infection (OBI) is characterized by the persistence of HBV DNA in liver and/or serum without detectable hepatitis B virus surface antigen (HBsAg) [1]. In most cases, the level of HBV DNA in serum is extremely low (<200 IU/mL). Over the past three decades, the prevalence of OBI and its potential clinical impacts have been intensively discussed in the setting of blood transfusion, liver transplantation, immunosuppressive conditions, etc [2]. In recent years, however, varying proportions of infants born to HBsAg-positive mothers have been diagnosed with OBI despite immunization against HBV, raising concerns that hepatitis B vaccine (HepB) may be ineffective for preventing OBI acquired from HBV mother-to-child transmission (MTCT) [3–6]. Intriguingly, the vast majority of the reported OBI-positive infants achieved protective levels of antibody to hepatitis B virus surface antigen (anti-HBs), without positivity for antibody to hepatitis B virus core antigen (anti-HBc). The maintenance of this cryptic condition remains elusive, mostly due to the lack of serial follow-ups of OBI-positive infants from a prospective birth cohort in previous studies. To elucidate this perplexity, in this study, we prospectively followed up a birth cohort of immunized neonates born to HBsAg-positive mothers at 12, 24 and 36 months of age, after being diagnosed with OBI at 7 months of age, one month after the completion of primary HBV vaccination.

## Materials and Methods

### Study participants

Pregnant women recruited from community population in Henan and Jiangsu province underwent HBsAg screening from August 2009 to June 2011. Those found to be HBsAg-positive in the preliminary screening were further examined for HBV serological markers and HBV DNA before labor at 37 weeks of gestation. The inclusion criteria for pregnant women were: ①HBsAg-positive; ②normal alanine aminotransferase (ALT) level; ③normal total bilirubin (TB) level; ④antiviral-naïve; ⑤without co-infection with hepatitis A virus, hepatitis C virus, hepatitis D virus, hepatitis E virus or human immunodeficiency virus; ⑥without any pregnancy complication. The inclusion criteria for newborns were: ①full-term; ②APGAR score≥7 at 1 min; ③birth weight ≥2,500 g; ④normal body temperature; ⑤normal jaundice index. The exclusion criteria for newborns were: ①congenital abnormality; ②neonatal acute infection; ③developmental disorder; ④family history of nervous system disease, coagulation disorder, immune dysfunction and allergy to vaccine components. Finally, a total of 158 mother-child pairs were enrolled in this study. Three-dose recombinant yeast-derived HepB were given to the enrolled neonates intramuscularly in the upper arm at 0 (within 12 hours of birth), 1 and 6 months. A birth dose of hepatitis B immunoglobulin (HBIG) was administered in the contralateral arm within 12 hours after birth. HepB (10μg/0.5mL; Kangtai Biological Products Co. Ltd., Shenzhen, China) and HBIG (100IU/1.0mL; Hualan Biological Engineering Inc., Xinxiang, China) were stored at 2–8°C until use. At 7 months of age, sera were collected from 158 neonates. Those diagnosed with OBI at 7 months of age were serially followed up at 12, 24 and 36 months of age. Sera were stored at -70°C until use. Repeated freezing and thawing were avoided. This study was approved by the Ethic Committee of Peking University Health Science Center and written informed consents were signed by parents of each enrolled neonate.

### Assays of HBV serological markers

HBV serological markers were determined by Abbott Architect i2000SR analyzer (Abbott Diagnostic, Chicago, IL, USA) on the basis of chemiluminiscent microparticle immunoassay (CMIA) [7]. Detection range of the HBsAg assay was 0.05–250 IU/mL. Sera were manually diluted 1:20 or 1:500 before further testing if HBsAg levels were higher than 250 IU/mL. Anti-HBs levels equal to or greater than 10 mIU/mL were defined as positive. Assay results for hepatitis B virus e antigen (HBeAg) and anti-HBc were reported as the ratio of sample relative light unit (RLU) to cut off RLU (S/CO), and S/CO values lower than 1.0 were considered negative.

### Quantitation of serum HBV DNA

HBV DNA was quantitated by Abbott m2000 (m2000sp+m2000rt) system using highly sensitive real-time PCR assay (Abbott Molecular, IL, USA) [8], the lower detection limit of which was 1.18 log IU/mL (15 IU/mL, 51 copies/mL). Negativity for HBV DNA was determined by assays results reported as “not detected” or “<1.18 log IU/mL”. All procedures were performed with cautions to avoid cross-contamination.

### Nested polymerase chain reaction (nPCR) and direct sequencing

HBV DNA extracted by Abbott m2000sp system was used for amplification of HBV RT, PreS and Core regions by nPCR. RT and Pre-S regions were amplified as previously described [9]. PCR primers and conditions for the amplification of Core region were shown in S1 Table and S2 Table. Amplification products underwent direct sequencing. Sequences were analyzed by Mega 5 and BLAST.

### HBV genotyping

HBV genotyping was performed by an nPCR method established in our laboratory previously [10], using HBV DNA extracted by Abbott m2000sp system.

### Definitions

The diagnosis criteria for OBI acquired from HBV MTCT in immunized neonates of HBsAg-positive mothers were being HBsAg-negative and HBV DNA-positive at 7 months of age, one month after a full course of primary HBV vaccination. Anti-HBs titers of 10–100 mIU/mL, 100–1000 mIU/mL and ≥1000 mIU/mL were defined as low, medium and high levels, respectively.

### Statistical analyses

Categorical variables were demonstrated as % (m/n) and examined by Chi square/Fisher's exact test. Continuous data were expressed as median values (ranges) and tested by Mann-Whitney *U*-test. Geometric mean concentrations (GMCs) with 95% confidence intervals (CIs) were calculated for anti-HBs levels. Data analyses were done by SPSS 17.0 statistical package. All *p* values were two-tailed and a *p* value<0.05 was considered statistically significant.

## Results

### Baseline characteristics

The median age of the pregnant women was 25.0 (18.7–41.0) years old. Among them, 94 (59.5%) were HBeAg-positive, with significantly higher HBV DNA levels and HBsAg titers than their HBeAg-negative counterparts (8.19 log IU/mL *vs*. 2.84 log IU/mL, *p*<0.001; 4.44 log IU/mL *vs*. 3.23 log IU/mL, *p<*0.001). Genotype C was predominant in both HBeAg-positive and HBeAg-negative pregnant women (Table 1).

**Table 1:** Baseline characteristics of pregnant women.

	Total	HBeAg (+)	HBeAg (-)	*p*-Value ^a^
Number	158	94 (59.5%)	64 (40.5%)	
Age (years)	25.0 (18.7–41.0)	23.0 (19.0–41.0)	27.2 (18.7–40.0)	<0.001
HBsAg (log IU/mL)	4.02 (-0.03–4.90)	4.44 (2.66–4.90)	3.23 (-0.03–4.27)	<0.001
HBV DNA (log IU/mL)	6.37 (0.00–9.11)	8.19 (2.05–9.11)	2.84 (0.00–7.09)	<0.001
Genotype				0.005
B	22 (16.7%)	20 (23.5%)	2 (4.3%)	
C	109 (82.6%)	64 (75.3%)	45 (95.7%)	
D	1 (0.7%)	1 (1.2%)	0 (0.0%)	
Unknown ^b^	26	9	17	

Continuous variables were expressed as median values (ranges).

HBV, hepatitis B virus; HBsAg, hepatitis B virus surface antigen; HBeAg, hepatitis B virus e antigen.

^a^ p-Values represented comparisons between HBeAg-positive and HBeAg-negative pregnant women; statistical differences were evaluated by Mann-Whitney U-test or Chi square/Fisher's exact test.

^b^ HBV genotypes were successfully identified in 132 mothers. In the other 26, genotyping was infeasible due to insufficient or lower levels of HBV DNA.

All the neonates (90 males, 68 females) were HBsAg-negative and anti-HBs-positive at 7 months of age. Low, medium and high levels of anti-HBs were achieved in 17.1% (27/158), 44.9% (71/158) and 38.0% (60/158) of them, respectively, with an anti-HBs GMC of 619.3 (485.4–790.1) mIU/mL. Thirty-two (20.3%) (19 males, 13 females) were diagnosed with OBI, with a median HBV DNA level of 1.97 (1.20–3.71) log IU/mL. Of them, the proportions mounting low, medium and high levels of anti-HBs were 18.8% (6/32), 46.9% (15/32) and 34.4% (11/32), respectively, with an anti-HBs GMC of 541.5 (336.1–872.4) mIU/mL.

### Homology and phylogenetic comparisons of mother-child HBV pairs

**Fig 1:**
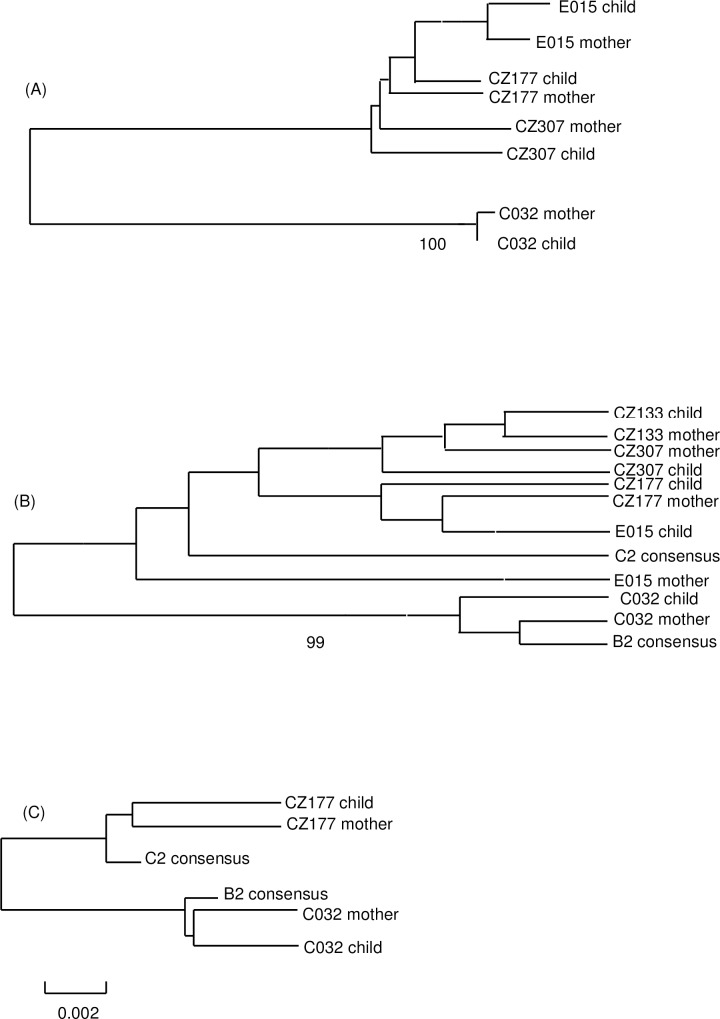
Phylogenetic trees of mother-child HBV pairs. (A) phylogenetic tree of RT region; (B) phylogenetic tree of Pre-S region; (C) phylogenetic tree of Core region.

Sequencing data were available for 5 mother-child pairs. Amplification followed by direct sequencing were infeasible for the others due to insufficient or lower levels of HBV DNA. HBV showed close homology and phylogenetic relationships for mother-child pairs (S3 Table, Fig 1). Genetic distances were estimated to be 0.002–0.016, 0.006–0.015 and 0.011–0.015 for mother-child pair-wise RT, Pre-S and Core regions, respectively. By contrast, genetic distances between all the available RT, Pre-S and Core fragments were 0.052, 0.056 and 0.025, respectively. With RT, Pre-S and Core subgenomes combined together, the estimated genetic distances for mother-child HBV pairs were 0.006–0.016. And the corresponding genetic distance between all the available integrated fragments was 0.051.

Sequences of S region were obtained in 4 neonates. The most common immune escape mutation, G145R, was found in none. RT region was successfully sequenced in 4 mother-child pairs. Drug-resistant variants were not discovered. Five neonates carried a total of 10 amino acid (aa) substitutions in Pre-S region, 5 of which existed as quasispecies. Importantly, 3 mutations (S101L, L165F, S96S/A) located in known T-/B-cell epitopes in Pre-S region. Identical aa substitutions in at least one HBV subgenome were found for 4 (80.0%) mother-child pairs (Table 2).

**Table 2:** Mutation spectra for mother-child HBV pairs.

	S	RT	Pre-S	Core
CZ133-Mother	/	/	None	/
CZ133-Child	/	/	A91V, S101L, R135K	/
CZ177-Mother	T5A	N13S, **L269I**	**V90A**	None
CZ177-Child	None	S256C, **L269I**	**V90V/A**, S96S/A, P173P/Q	V114I, P159T
E015-Mother	None	S256C, **L269I**	A158V	/
E015-Child	None	**L269I**, K333Q	None	/
CZ307-Mother	I168T, V184A	G127W, L267R, L269I, Q316H	**V90A**	/
CZ307-Child	W74L, S204N	None	I184I/M, **V90A**	/
C032-Mother	**F200Y**, **M213T**	**L336S**, **N337T**	L84I	None
C032-Child	T45A, **F200Y**, **M213T**	S50F, N53S, N134N/D, **L336S**, **N337T**	A62A/T, L165F	None

Characters in bold referred to amino acid substitutions identical for mother-child HBV pairs. /, not successfully sequenced due to insufficient or lower levels of HBV DNA.

### Dynamic changes of HBV markers in OBI-positive neonates during follow-ups

Of the 32 OBI-positive neonates at 7 months of age, 32 were followed up to 12 months of age, 32 to 24 months of age and 26 to 36 months of age. The main reasons for dropping out were unwillingness to undergo puncture in neonate, long distance from hospital and invalid phone number.

Among them, 25.0% (8/32), 21.9% (7/32) and 7.7% (2/26) were positive for HBV DNA at 12, 24 and 36 months of age, respectively. The median level of HBV DNA was 1.81 (1.28–2.91) log IU/mL at 12 months of age, 1.94 (1.23–2.58) log IU/mL at 24 months of age and 1.74 (1.59–1.89) log IU/mL at 36 months of age. HBV DNA disappeared at one of the follow-up time points in 31 neonates, of whom, 24 at 12 months of age, 5 at 24 months of age and 2 at 36 months of age. However, HBV DNA rebounded to low levels thereafter in 6 of the 31, 4 at 24 months of age and 2 at 36 months of age. HBV DNA persisted at low levels during follow-ups in the other one neonate apart from the above 31.

Anti-HBs was positive in 93.8% (30/32) at 12 months of age, 70.0% (21/30) at 24 months of age and 82.4% (14/17) at 36 months of age. Two neonates at 24 months of age and 9 at 36 months of age, who had received booster doses of vaccine, were excluded from analysis. Low, medium and high levels of anti-HBs were found in 16.7% (5/30), 66.7% (20/30), and 16.7% (5/30) at 12 months of age, 66.7% (14/21), 33.3% (7/21) and 0.0% (0/21) at 24 months of age, and 64.2% (9/14), 35.7% (5/14) and 0.0% (0/14) at 36 months of age, respectively. Anti-HBs GMC was 239.2 (127.1–450.2) mIU/mL at 12 months of age, 26.7 (8.4–32.5) mIU/mL at 24 months of age and 34.3 (17.6–67.1) mIU/mL at 36 months of age.

All remained negative for HBsAg throughout follow-ups to 36 months of age.

Anti-HBc disappeared and remained negative thereafter in 30 neonates, of whom, 3 at 7 months of age, 22 at 12 months of age and 5 at 24 months of age. Only 2 (6.3%) neonates were positive for anti-HBc after 24 months of age.

### Risk factor

**Fig 2:**
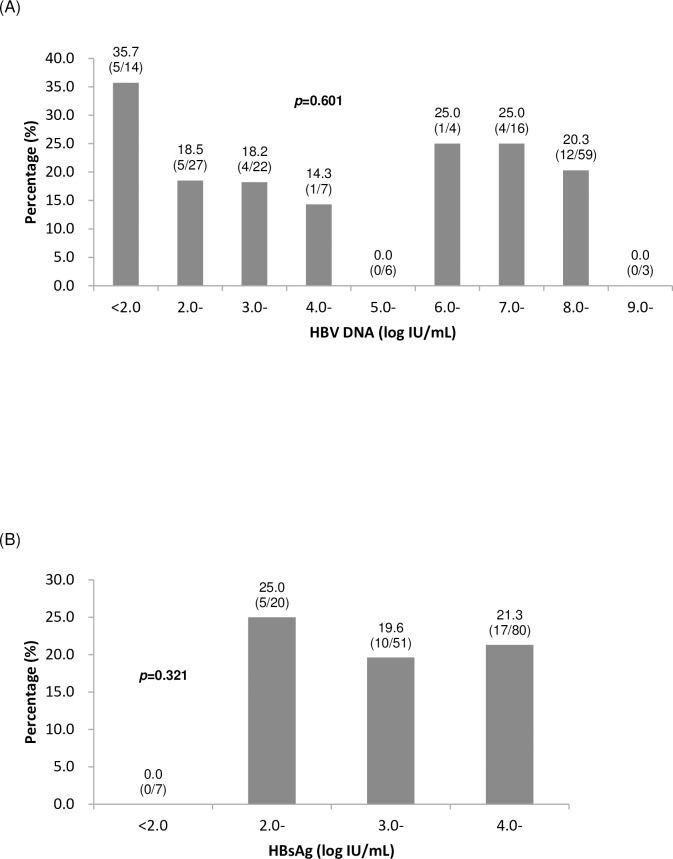
The occurrence of OBI stratified by maternal HBV DNA levels and HBsAg titers. (A) stratified by HBV DNA levels; (B) stratified by HBsAg titers.

Potential factors in both mothers and neonates were analyzed to investigate the possible risk factor for OBI in neonates born to HBsAg-positive mothers despite immunization (Table 3). The only factor with statistical significance was the first dose of vaccine series >6 hours of birth. A much lower proportion of OBI-positive neonates received the first dose of vaccine series within 6 hours after birth compared with their OBI-negative counterparts (59.4% *vs*. 83.3%, *p* = 0.003). Timely administration of the first dose of vaccine decline the rate of OBI from 38.2% (13/34) to 15.3% (19/124) (*p* = 0.003). However, detectable HBV DNA during follow-up was not attributed to the first dose of vaccine series after 6 hours of birth (5/13, 38.5% *vs*. 8/19, 42.1%, *p* = 0.837). No significant differences were revealed with regard to the occurrence of OBI when further stratifying maternal HBV DNA levels and HBsAg titers (Fig 2).

**Table 3:** Potential risk factors for OBI in immunized neonates of HBsAg-positive mothers.

Factors	Total (n = 158)	OBI(+) (n = 32)	OBI(-) (n = 126)	*p*-Value ^a^
Neonate				
Gender (Male: Female)	90:68	19:13	71:55	0.758
Anti-HBc positivity rate (%) ^b^	73.4 (91/124)	83.3 (15/18)	71.7 (76/106)	0.457
Anti-HBs (mIU/mL) (%)				0.889
10–100	17.1 (27/158)	18.8 (6/32)	16.7 (21/126)	
100–1000	44.9 (71/158)	46.9 (15/32)	44.4 (56/126)	
≥1000	38.0 (60/158)	34.4 (11/32)	38.9 (49/126)	
HBIG (IU) (%)				0.477
100	50.6 (80/158)	56.3 (18/32)	49.2 (62/126)	
200	49.4 (78/158)	43.8 (14/32)	50.8 (64/126)	
First vaccine dose within 6 hours (%)	78.5 (124/158)	59.4 (19/32)	83.3 (105/126)	**0.003**
Mother				
HBsAg (log IU/mL)	4.02 (-0.03–4.90)	4.05 (2.10–4.81)	4.01 (-0.03–4.90)	0.990
HBeAg positivity rate (%)	59.5 (94/158)	50.0 (16/32)	61.9 (78/126)	0.221
HBV DNA (log IU/mL)	6.37 (0.00–9.11)	6.83 (1.29–8.90)	6.17 (0.00–9.11)	0.496
Parturition manner (Caesarean: Vaginal)	97:61	22:10	75:51	0.338
Feeding pattern (Artificial: Breast ^c^)	113:45	22:10	91:35	0.698

Continuous variables were expressed as median values (ranges).

HBV, hepatitis B virus; HBsAg, hepatitis B virus surface antigen; HBeAg, hepatitis B virus e antigen; HBIG, hepatitis B immunoglobulin; anti-HBs, antibody to hepatitis B virus surface antigen; anti-HBc, antibody to hepatitis B virus core antigen.

^a^ p-Values represented comparisons between OBI-positive and OBI-negative neonates at 7 months of age; statistical differences were evaluated by Mann-Whitney U-test or Chi square/Fisher's exact test.

^b^ Assay results of anti-HBc were unknown in 34 neonates due to insufficient sera.

^c^ Breast-feeding included mixed feeding.

## Discussion

In recent years, OBI has been found in infants born to HBsAg-positive mothers despite immunization. However, the reported rates of OBI in this subgroup varied greatly [3–6], from 1.6% to 64.0%, possibly due to differential inclusion criteria for pregnant women and their newborns, sample sizes, immunization procedures, sensitivity and specificity of the adopted detection methods, etc. We enrolled a birth cohort of 158 neonates born to antiviral-naive HBsAg-positive mothers in this study. Notably, previous studies were almost exclusively cross-sectional without data from a prospective birth cohort. The reported cases did not necessarily indicate OBI acquired from HBV MTCT owing to immunoprophylaxis failures. Horizontal transmission could not be totally excluded. OBI was diagnosed at 7 months of age, one month after the completion of primary HBV immunization. The Abbott m2000 system with highly sensitive real-time PCR assay, the accuracy, sensitivity and repeatability of which were widely approved, was exploited to detect serum HBV DNA in the diagnosis of OBI [8]. As a result, thirty-two (20.3%) neonates were diagnosed with OBI. Importantly, homology comparisons of mother-child HBV pairs rarely done in previous reports were carried out in this study to rule out potential cross-contamination [11]. Close homology and phylogenetic relationships were revealed for mother-child HBV pairs, ascertaining the diagnosis to be true OBI cases acquired from MTCT rather than occult cross-contamination. Consistent with existing findings that 67.0% to 100.0% of the OBI-positive infants of HBsAg-positive mothers were positive for anti-HBs [3–6], all such neonates in our study also responded to primary HBV vaccination.

Then we observed the maintenance of this status through serial follow-ups at 12, 24 and 36 months of age. HBV DNA declined to undetectable levels in 31 (96.9%, 31/32) neonates at one of the follow-up points, however, rebounded thereafter to low levels in 6 (18.8%, 6/32) of them. In other words, HBV DNA disappeared at one of the follow-up points and remained negative thereafter in 25 (78.1%, 25/32) neonates, 19 at 12 months of age, 4 at 24 months of age and 2 at 36 months of age. In agreement with our findings, the study by Sadeghi et al. discovered that HBV DNA disappeared in 94% (16/17) of the OBI-positive children 36 months after the initial sampling. HBV DNA was also undetectable in the still OBI-positive child eighteen months later [12]. They therefore claimed the clearance of HBV in those children. However, considering that OBI is characterized by the persistence of intrahepatic HBV covalently closed circular DNA (cccDNA), the golden standard for the determination of HBV clearance should be liver biopsy, which is not indicated in neonates due to ethical reasons [13]. Indeed, the clearance of HBV as evidenced from the disappearance of HBV DNA from sera might not always be reliable, as shown in our study that though HBV DNA declined to undetectable levels at one of the follow-up points, rebounds to low levels were observed thereafter. We assume that HBV may be under intact immune control, with circulating HBV DNA at considerably low levels, frequently below the lower detection limits of commercial assays. HBV DNA may disappear from sera at times, however, HBV cccDNA long persists in hepatocytes, resulting in the pattern of intermittent viraemia demonstrated in this study. Therefore, serial samplings of serum HBV DNA as an alternate of liver biopsy may be used to determine the status of OBI in neonates. Intriguingly, phases of absent viraemia alternating with phases of extremely low but detectable HBV DNA have also been observed with adult patients with OBI [13]. Besides, though anti-HBs gradually declined, even to unprotected levels in this study, all including the one (3.1%, 1/32) neonate persistently positive for HBV DNA during follow-ups showed no evidence of HBsAg-positive infection, the consequence of vertical HBV infection in the vast majority of newborns. All these results suggested the ability of neonates to prime an immune reaction, innate or adaptive, to control the replication of HBV at extremely low levels [14,15]. Therefore, neonatal immune system may defy the simple categorization of being defective. Moreover, the efficacy of primary HBV vaccination in OBI-positive neonates born to HBsAg-positive mothers challenged immune tolerance as being inevitable after exposure to HBV in neonates [14,15]. Responses to booster doses of vaccine in 11 neonates in this study also verified this notion (S4 Table). Vice versa, the importance of vaccination against HBV for utmost protection was emphasized. Possible clinical outcomes, if any, warranted prospective evaluation from serial follow-ups in a long-term perspective. Though HBV is strongly suppressed by immune system, this suppression may not be absolute. Residual HBV may persist over time and possibly leads to development of overt HBV infection under circumstances [13].

S-escape mutants, such as G145R, unrecognized by diagnostic kits, may account for the absence of HBsAg observed in some cases [13]. In this study, common immune escape mutations in S region were not discovered, ruling out the possibility of “false” OBI. Besides, the Abbott assay may detect HBsAg produced by multiple S-escape mutants, including G145R. On the other hand, mutations frequently existed as quasispecies in Pre-S region, implicating that traces of HBV genome might be under strong immune pressure in the status of OBI. Friedt et al. found that the evolutionary rates of dominant variants could reach 6–43 bases in the complete genome of HBV within the first six months in newborns acquiring HBV from their HBsAg-positive mothers [16]. In this study, pair-wise genetic distances between mother-child HBV sequences were 0.006–0.016, with 3–33 bases in discrepancy, suggesting the evolution of dominant strains, or the changes of quasispecies constitutions, once transmitted from mothers to neonates. However, more studies are needed to investigate the immunological and viral profiles after occult or chronic infection with HBV acquired from vertical transmission in neonates born to HBsAg-positive mothers.

In previous studies, 50.0% to 100.0% of the OBI-positive infants of HBsAg-positive mothers demonstrated negativity for anti-HBc [3–6,12]. It seems to deviate from the consensus that anti-HBc is the lifelong marker of past exposure to or present infection with HBV [17]. One thing to be noted is that positivity for anti-HBc before 24 months of age was largely from transplacental transfer of the maternal antibody [18]. Therefore, the presence of anti-HBc after 24 months of age could better reflect neonatal immune response and memory against HBV. However, in this study, only two (6.3%) neonates were positive for anti-HBc after 24 months of age. Similarly, Sadeghi et al. found that 23.8% (5/21) of the OBI-positive infants of HBsAg-positive mothers were positive for anti-HBc in the initial sampling [3]. The incidence was likely to be even lower since 80.0% (4/5) of them were under 24 months of age. Indeed, at the second sampling 36 months later, anti-HBc disappeared in 80.0% (4/5) of them, leading to a final anti-HBc positivity rate of about 5.9% (1/17) [12]. We assume that strong immune pressure derived from passive-active immunization against HBV could allow the establishment of a low-dose HBV infection without sufficient antigens to stimulate and maintain the maturation of immune memory, which was supported by discoveries in woodchuck infection model that exposure to low levels of HBV DNA (<10^3^ virions) might develop into anti-HBc seronegative infection [19]. Or rather, the levels of anti-HBc were simply too low to be detected by commercial assays [17] and long-term follow-ups might be critical to decide whether anti-HBc was negative in such subjects.

Possible risk factors for OBI in immunized infants of HBsAg-positive mothers remain uncertain in previous studies [3–6,12]. Maternal HBeAg and HBV DNA at high levels in circulation might contribute to OBI status in their neonates after immunization [4,5]. However, no significant differences with regard to the occurrence of OBI in immunized neonates were revealed when further stratified by maternal HBV DNA levels and HBsAg titers in this study. The only risk factor with statistical significance was the first dose of vaccine series >6 hours of birth. We assume that OBI may be acquired from low-dose exposure to maternal HBV and thus may not be always related to high levels of maternal viral load like overt HBV infection. Timely administration of the first vaccine dose would probably induce HBV-specific T- and B-cell responses as early as possible to prevent HBV infection most efficiently.

## Supporting Information

S1 Table Primers for amplification of Core region. (DOCX)

S2 Table PCR mix and PCR parameters for amplification of Core region. (DOCX)

S3 Table Homology comparisons of mother-child HBV pairs. -, data not available.
(DOCX)

S4 Table Follow-ups of OBI-positive neonates at 12, 24 and 36 months of age. /, data unavailable due to insufficient sera; P, positive; N, negative.
(DOCX)

## References

[1] Raimondo G, Allain JP, Brunetto MR, Buendia MA, Chen DS, Colombo M, et al Statements from the Taormina expert meeting on occult hepatitis B virus infection. J Hepatol. 2008; 49: 652–7. 10.1016/j.jhep.2008.07.014 18715666

[2] Raimondo G, Caccamo G, Filomia R, Pollicino T. Occult HBV infection. Semin Immunopathol. 2013; 35: 39–52. 10.1007/s00281-012-0327-7 22829332 PMC3540364

[3] Shahmoradi S, Yahyapour Y, Mahmoodi M, Alavian SM, Fazeli Z, Jazayeri SM. High prevalence of occult hepatitis B virus infection in children born to HBsAg-positive mothers despite prophylaxis with hepatitis B vaccination and HBIG. J Hepatol. 2012; 57: 515–21. 10.1016/j.jhep.2012.04.021 22617152

[4] Pande C, Sarin SK, Patra S, Kumar A, Mishra S, Srivastava S, et al Hepatitis B vaccination with or without hepatitis B immunoglobulin at birth to babies born to HBsAg-positive mothers prevents overt HBV transmission but may not prevent occult HBV infection in babies: a randomized controlled trial. J Viral Hepat. 2013; 20: 801–10. 10.1111/jvh.12102 24168259

[5] Su H, Zhang Y, Xu D, Wang B, Zhang L, Li D, et al Occult hepatitis B virus infection in anti-HBs-positive infants born to HBsAg-positive mothers in China. PLoS One. 2013; 8: e70768 10.1371/journal.pone.0070768 23951004 PMC3741317

[6] Foaud H, Maklad S, Mahmoud F, EI-Karaksy H. Occult hepatitis B virus infection in children born to HBsAg-positive mothers after neonatal passive-active immunoprophylaxis. Infection. 2015; 43: 307–14. 10.1007/s15010-015-0733-6 25665956

[7] Deguchi M, Yamashita N, Kagita M, Asari S, Iwatani Y, Tsuchida T, et al. Quantitation of hepatitis B surface antigen by an automated chemiluminescent microparticle immunoassay. J Virol Methods. 2004; 115: 217–22. 14667538 10.1016/j.jviromet.2003.10.002

[8] Ciotti M, Marcuccilli F, Guenci T, Prignano MG, Perno CF. Evaluation of the Abbott RealTime HBV DNA assay and comparison to the Cobas AmpliPrep/Cobas TaqMan 48 assay in monitoring patients with chronic cases of hepatitis B. J Clin Microbiol. 2008; 46: 1517–9. 10.1128/JCM.02046-07 18272717 PMC2292927

[9] Nie JJ, Sun KX, Li J, Wang J, Jin H, Wang L, et al. A type-specific nested PCR assay established and applied for investigation of HBV genotype and subgenotype in Chinese patients with chronic HBV infection. Virol J. 2012; 9: 121 10.1186/1743-422X-9-121 22716091 PMC3477104

[10] Jin H, Wang J, Yan L, Nie JJ, Li J, Zhuang H. Estabilshment of a nested PCR to identify hepatitis B virus genotypes A-D and subgenotypes B1, B2, C1 and C2. Chin J Epidemiol. 2008; 29: 1235–9. 19173971

[11] Zhou YH. Occult hepatitis B virus infection, or occult cross-contamination, in children of HBsAg-positive mothers. J Viral Hepat. 2016; 10.1111/jvh.12559 27333952

[12] Sadeghi A, Yahyapour Y, Poortahmasebi V, Shahmoradi S, Roggendorf M, Karimzadeh H, et al Clearance of HBV DNA in immunized children born to HBsAg-positive mothers, years after being diagnosed with occult HBV infection. J Viral Hepat. 2016; 23: 282–5. 10.1111/jvh.12490 26598112

[13] Pollicino T, Raimondo G. Occult Hepatitis B Infection. J Hepatol. 2014; 61: 688–9. 10.1016/j.jhep.2014.04.036 24976111

[14] Bertoletti Antonio, Kennedy Patrick T. The immune tolerant phase of chronic HBV infection: new perspectives on an old concept. Cellular & Molecular Immunology. 2010; 12: 258–263. 10.1038/cmi.2014.79 PMC4654319 25176526

[15] Bertoletti Antonio, Hong Michelle. Age-dependent immune events during HBV infection from birth to adulthood: an alternative interpretation. Front Immunol. 2014; 5: 441 10.3389/fimmu.2014.00441 25295036 PMC4172010

[16] Friedt M, Gerner P, Wintermeyer P, Wirth S. Complete hepatitis B virus genome analysis in HBsAg positive mothers and their infants with fulminant hepatitis B. BMC Gastroenterol 2004; 4: 11 10.1186/1471-230X-4-11 15186503 PMC425580

[17] Yeh CT, Lai MW. Eliminating hepatitis B virus through neonatal vaccination: Can we make it? J Hepatol. 2012; 57: 484–5. 10.1016/j.jhep.2012.06.003 22683335

[18] Wang JS, Chen H, Zhu QR. Transformation of hepatitis B serologic markers in babies born to hepatitis B surface antigen positive mothers. World J Gastroenterol. 2005; 11: 3582–5. 10.3748/wjg.v11.i23.3582 15962380 PMC4315966

[19] Mulrooney-Cousins PM, Michalak TI. Persistent occult hepatitis B virus infection: experimental findings and clinical implications. World J Gastroenterol. 2007; 13: 5682–6. 10.3748/wjg.v13.i43.5682 17963292 PMC4171252

