# Supplementary material for: Occult HBV Infection in Immunized Neonates Born to HBsAg-Positive Mothers: A Prospective and Follow-Up Study
Source: PLoS One. 2016 Nov 11;11(11):e0166317. doi: 10.1371/journal.pone.0166317 (PMC5106040; doi:10.1371/journal.pone.0166317)
Supplement: S1 Table — (DOCX) [file pone.0166317.s001.docx]

| Primer | Sequence | Position (nt) |
| --- | --- | --- |
| C1 | 5’-CAT AAG AGG ACT CTT GGA CT-3’ | 1653–1672 |
| C6 | 5’-TCT CCT GTT TTC ATC AAC TGT AA-3’ | 2624-2602 |
| C3 | 5’-GAG GAC TTG GGG GAG GAG ATT-3’ | 1734–1754 |
| C8 | 5’-CAG GTA CAG TAG AAG AAT AAA GCC-3’ | 2511-2488 |
