# Supplementary material for: Occult HBV Infection in Immunized Neonates Born to HBsAg-Positive Mothers: A Prospective and Follow-Up Study
Source: PLoS One. 2016 Nov 11;11(11):e0166317. doi: 10.1371/journal.pone.0166317 (PMC5106040; doi:10.1371/journal.pone.0166317)
Supplement: S2 Table — (DOCX) [file pone.0166317.s002.docx]

| PCR Mix for 1^st^ round |  | PCR Parameters |
| --- | --- | --- |
| 10×Ex Buffer (Mg^2+^) | 2.0μL | 95℃ 5min |
| dNTPs (2.5mM each) | 0.4μL | 95℃ 30s, 54℃ 30s, 72℃ 45s, for 40 cycles |
| C1 (10uM) | 0.6μL | 72℃ 7min |
| C6 (10uM) | 0.6μL | 4℃ for conservation |
| Ex Taq DNA polymerase (TaKaRa, Dalian, China) | 0.3μL |  |
| ddH_2_O | 11.1μL |  |
| Extracted HBV DNA | 5.0μL |  |
| PCR Mix for 2^nd^ round |  | PCR Parameters |
| 10×Ex Buffer (Mg^2+^) | 3.0μL | 95℃ 5min |
| dNTPs (2.5mM each) | 0.6μL | 95℃ 30s, 54℃ 30s, 72℃ 45s, for 40 cycles |
| C3 (10uM) | 0.9μL | 72℃ 7min |
| C8 (10uM) | 0.9μL | 4℃ for conservation |
| Ex Taq DNA polymerase (TaKaRa, Dalian, China) | 0.4μL |  |
| ddH_2_O | 23.3μL |  |
| PCR-amplified products from the first round | 1.0μL |  |
