# Supplementary material for: Occult HBV Infection in Immunized Neonates Born to HBsAg-Positive Mothers: A Prospective and Follow-Up Study
Source: PLoS One. 2016 Nov 11;11(11):e0166317. doi: 10.1371/journal.pone.0166317 (PMC5106040; doi:10.1371/journal.pone.0166317)
Supplement: S3 Table — -, data not available. (DOCX) [file pone.0166317.s003.docx]

| Mother | Child | Homology | | |
| --- | --- | --- | --- | --- |
|  |  | RT (%) | Pre-S (%) | Core (%) |
| CZM133 | CZC133-1 | - | 98 | - |
| CZM177 | CZC177-1 | 99 | 97 | 99 |
| E015 | EX033-1 | 99 | 99 | - |
| CZM307 | CZC307-1 | 98 | 99 | - |
| C032 | CX005-1 | 99 | 98 | 99 |
