# Supplementary material for: Occult HBV Infection in Immunized Neonates Born to HBsAg-Positive Mothers: A Prospective and Follow-Up Study
Source: PLoS One. 2016 Nov 11;11(11):e0166317. doi: 10.1371/journal.pone.0166317 (PMC5106040; doi:10.1371/journal.pone.0166317)
Supplement: S4 Table — /, data unavailable due to insufficient sera; P, positive; N, negative. (DOCX) [file pone.0166317.s004.docx]

|  | 7 months of age | | | 12 months of age | | | 24 months of age | | | 36 months of age | | |
| --- | --- | --- | --- | --- | --- | --- | --- | --- | --- | --- | --- | --- |
|  | HBV DNA  (log IU/mL) | Anti-HBs  (mIU/mL) | Anti-HBc | HBV DNA  (log IU/mL) | Anti-HBs  (mIU/mL) | Anti-HBc | HBV DNA  (log IU/mL) | Anti-HBs  (mIU/mL) | Anti-HBc | HBV DNA  (log IU/mL) | Anti-HBs  (mIU/mL) | Anti-HBc |
| CZC093 | 1.20 | 64.12 | P | N | 9.9 | N | N | 1000.00 | N | N | 474.88 | N |
| CZC259 | 2.35 | 64.49 | / | N | 774.1 | N | N | 21.89 | P | N | 68.33 | P |
| CZC015 | 1.96 | 77.28 | / | N | 10.26 | / | N | 0.37 | N | N | 12.53 | N |
| CZC177 | 2.65 | 122.31 | / | N | 10.04 | N | N | 9.33 | N | N | 8.17 | N |
| CZC020 | 2.75 | 295.95 | / | N | 274.55 | / | N | 5.22 | N | N | 367.19 | N |
| CZC136 | 3.71 | 303.51 | N | N | 163.78 | N | N | 17.91 | N | N | 15.99 | N |
| CZC159 | 1.61 | 356.79 | / | N | 701.82 | N | N | 55.62 | N | / | / | / |
| CZC013 | 2.01 | 587.16 | / | N | 465.94 | N | N | 72.38 | N | N | 72.39 | N |
| CZC449 | 1.54 | 609.2 | P | N | 211.51 | N | N | 36.60 | N | N | 19.78 | N |
| CZC144 | 3.17 | 829.46 | / | N | 142.08 | N | N | 0.87 | N | / | / | / |
| FX091 | 1.24 | 870.82 | P | N | 439.27 | N | N | 27.09 | N | / | / | / |
| CZC049 | 2.36 | 913.68 | / | N | 715.73 | N | N | 7.12 | N | N | 92.20 | N |
| CX005 | 2.38 | 1000 | N | N | 320.3 | N | N | 41.64 | N | N | 39.21 | N |
| CZC028 | 1.59 | 1046.97 | / | N | 601.69 | / | N | 5.57 | N | N | 3004.95 | N |
| FX031 | 2.35 | 1060.58 | P | N | 1139.38 | P | N | 681.75 | P | N | 138.91 | P |
| CZC157 | 3.20 | 1204.49 | / | N | 950.82 | N | N | 74.77 | N | N | 45.92 | N |
| CX019 | 1.50 | 1991.14 | P | N | 352.56 | N | N | 51.23 | N | N | 12.14 | N |
| EX062 | 1.97 | 2414.49 | P | N | 962.9 | P | N | 405.2 | N | / | / | / |
| CZC054 | 1.83 | 3770.8 | / | N | 162.68 | N | N | 372.12 | N | N | 290.33 | N |
| TXC220 | 2.52 | 63.55 | P | N | 3.84 | N | 1.67 | 0.36 | N | N | 247.41 | N |
| CZC231 | 1.88 | 298.63 | / | N | 171.34 | N | 1.94 | 147.21 | N | N | 67.81 | N |
| CZC307 | 1.23 | 387.86 | P | N | 118.34 | N | 1.23 | 5.49 | N | N | 141.71 | N |
| EX089 | 2.35 | 1598.47 | P | N | 36.55 | N | N | 38.1 | N | 1.59 | 40.91 | N |
| CX008 | 1.71 | 1644.29 | P | N | 4794.68 | N | 1.94 | 783.45 | N | N | 225.27 | N |
| CZC193 | 1.37 | 42.16 | P | 1.51 | 911.86 | N | N | 52.61 | N | N | 806.71 | N |
| DX022 | 2.01 | 367.64 | P | 1.28 | 2152.51 | N | N | 11.48 | N | N | 6.1 | N |
| CZC003 | 2.91 | 636.87 | / | 2.58 | 77.85 | / | N | 113.18 | N | N | 2786.78 | N |
| FX129 | 1.72 | 2634.48 | P | 2.91 | 1596.1 | P | N | 228.84 | N | / | / | / |
| CZC037 | 1.96 | 1329 | / | 1.91 | 283.99 | N | N | 8.41 | N | 1.89 | 4.63 | N |
| CZC345 | 1.25 | 785.82 | P | 1.57 | 492.68 | P | 1.43 | 15.07 | N | N | 1033.17 | N |
| EX033 | 1.39 | 8208.73 | N | 1.85 | 1407.36 | N | 2.21 | 603.76 | N | N | 158.99 | N |
| CZC133 | 2.70 | 96.33 | P | 2.16 | 16 | N | 2.58 | 14.81 | N | / | / | / |
